# Patterns of medicinal cannabis prescriptions in diverse patient populations: a retrospective analysis

**DOI:** 10.1186/s42238-025-00307-6

**Published:** 2025-07-12

**Authors:** Omer Edni, Eviatar Naamany, Shimon Izhakian, Shachaf Shiber

**Affiliations:** 1 Pulmonary Division, Rabin Medical Center, Petach Tikva, Israel; 2 Faculty of Medical and Health Sciences, Tel Aviv University, Tel Aviv, Israel; 3 Department of Emergency Medicine, Rabin Medical Center, Beilinson Campus, Petah Tikva, 49100 Israel

**Keywords:** Medicinal Cannabis, Chronic disease, THC/CBD ratio, Sex medicine

## Abstract

**Introduction:**

The use of medicinal cannabis is increasing worldwide and has applications in managing a wide range of conditions, including neurological, rheumatological, and gastrointestinal diseases. Despite its growing prevalence, there are limited data on patterns of cannabis prescription across varying disease groups and demographic subgroups.

**Methods:**

This retrospective observational cohort study analysed cannabis usage patterns among 263 patients from the cannabis user clinic at Rabin Medical center (RMC), a tertiary hospital in Israel. To minimise the inclusion of recreational cannabis users, only patients aged 30 years and older were included. Patients were categorised into three groups based on their primary medical condition: neurological (*n* = 63), rheumatological (*n* = 106), and gastrointestinal (*n* = 94). Data collected included: demographic information, cannabis dosage, Tetrahydrocannabinol (THC) content, cultivated variety preference (sativa vs. Indica), and method of consumption (smoking vs. oil). Statistical analyses were conducted using ANOVA, Kruskal-Wallis, chi-square, and t-tests to compare cannabis prescription patterns between disease and demographic groups.

**Results:**

Significant differences in cannabis prescription patterns were observed across disease groups. Patients with gastrointestinal conditions were prescribed the highest mean monthly cannabis dose (22.26 ± 13.60 g), while those with neurological conditions had the highest oil consumption (31.75%). Sex-based differences were notable, with male patients being prescribed significantly higher doses of cannabis (25.48 ± 15.15 g) and higher THC content (14 ± 6.56%) compared to female patients (17.32 ± 9.93 g; THC: 11.39 ± 6.48%).

**Discussion:**

The study highlights variations in cannabis prescription patterns based on both medical conditions and demographic factors. Male patients received higher doses and THC-rich formulations, while patients with gastrointestinal conditions had the highest cannabis prescription overall. These findings suggest the need for individualised cannabis therapy based on patient characteristics and the specific condition being treated.

**Conclusion:**

Medicinal cannabis usage patterns vary significantly across disease and demographic groups. Personalised cannabis treatment plans, informed by both clinical and demographic factors, are essential to optimising patient outcomes. Further research is needed to develop more precise guidelines for prescribing medicinal cannabis.

## Introduction

The therapeutic use of cannabis and cannabis-derived substances has seen significant growth in recent years despite a relative scarcity of high-quality scientific evidence supporting its efficacy across various medical conditions (National Academies of Sciences, E. and et al. 2017). Cannabis-based products, often non-purified, primarily contain two key cannabinoids: cannabidiol (CBD) and delta-9-tetrahydrocannabinol (THC). CBD is known for its anti-inflammatory, neuroprotective, anxiolytic, and antipsychotic properties (Fernández-Ruiz et al. 2013), whereas THC, the primary psychoactive component, has been found effective in alleviating symptoms such as pain, nausea, and sleep disturbances (National Academies of Sciences, E. and et al. 2017; Vandrey et al. 2017). Together, these cannabinoids form the backbone of medicinal cannabis applications, with varying therapeutic implications depending on the condition being treated.

Medicinal cannabis is now being used to manage an increasing array of chronic conditions, including neuropathic pain, fibromyalgia, neurodegenerative diseases like Alzheimer’s and Parkinson’s, seizure disorders, and inflammatory diseases such as inflammatory bowel disease (IBD) (Hazekamp et al. 2013; Sexton et al. 2016; Troutt and DiDonato 2015; Aggarwal et al. 2018; Reinarman et al. 2011). In Israel, the licensing of medical cannabis began in 2007 under the regulation of the Ministry of Health. This licensing process, following a physician’s recommendation for approved indications and only after conventional treatments have been exhausted, links patients to specialised clinics for ongoing care and monitoring.

While the utilisation of medical cannabis continues to expand globally, registries tracking patient outcomes remain relatively new, and the number of well-designed observational or clinical studies is limited. Existing research, though insightful, has not adequately explored the differences in cannabis prescription patterns across various disease groups, demographic factors such as sex, or other demographic variables (Bar-Lev Schleider et al. 2022; Ware et al. 2004; Ogborne et al. 2000; Harris et al. 2000; Lake et al. 2020). 

For example, understanding the differential prescription of THC versus CBD among patient populations could provide valuable insights into treatment goals and patient preferences. Given the distinct pharmacological profiles of THC and CBD, especially THC’s psychotomimetic effects (Lawn et al. 2023), It is essential to examine whether specific patient populations are prescribed different cannabis formulations based on their medical conditions.

This study focuses on the characteristics of adult patients aged 30 and older who were licensed to use medical cannabis and were treated at the outpatient clinic of Rabin Medical Center. By investigating the epidemiology and usage patterns within this cohort, the study aims to shed light on the demographic and clinical factors influencing cannabis prescription patterns for various medical etiologies.

## Methods

### Study design, settings, and ethics

This single-centre, retrospective observational study was conducted at Rabin Medical Center, Beilinson Hospital, Israel, between 2021 and 2025. Ethical approval for this study was obtained from the Institutional Review Board of Rabin Medical Centre (Chairperson Prof. Ran Tur-Kaspa). This manuscript adheres to the STROBE statement (Elm et al. 2007). 

To analyse differences in medicinal cannabis prescription, patients were grouped based on two main factors: (1) primary medical indication for cannabis use and (2) demographic characteristics.

#### Indication groups

Patients were categorised into three primary disease groups based on their referral diagnosis:

- **Neurological conditions**: This group included patients with diagnoses such as chronic neuropathic pain, epilepsy, multiple sclerosis, Alzheimer’s disease, and Parkinson’s disease.
- **Rheumatological conditions**: Patients with conditions such as fibromyalgia, rheumatoid arthritis, and other Rheumatological conditions were included in this group.
- **Gastroenterological conditions**: Patients with inflammatory bowel disease (IBD), Crohn’s disease, and ulcerative colitis were grouped here.

These groups were selected to reflect the primary medical indications for which cannabis is commonly prescribed.

#### Demographic groups

In addition to medical indication, patients were differentiated based on the following demographic characteristics:

1. **Sex**: Male and female patients were analysed separately to identify any sex-based differences in cannabis prescription patterns.
2. **Marital status**: This was categorised as married, single, divorced, or widowed.

#### Cannabis prescription variables

For each group, we analysed the following cannabis prescription variables:

- **Monthly cannabis dosage (grams)**: Total amount of cannabis prescribed per month.
- **THC/CBD ratio**: Proportion of THC versus CBD in the prescribed cannabis products.
- **Method of consumption (oil vs. smoking)**: The form of cannabis that patients were using, either oil-based or inhaled via smoking. Regarding the choice of consumption method, we practice shared clinical decision-making with our patients. Patients who request smoking or oil methods (unless contraindicated) are usually prescribed their preferred method.

These grouping variables allowed us to examine differences in cannabis prescription patterns both within and across the medical and demographic categories. Statistical comparisons were performed to identify significant differences in cannabis prescription characteristics among these groups.

### Eligibility criteria

Patients were eligible for inclusion in the study if they met the following criteria:

1. Adult patients aged 30 years or older — this age threshold was selected to reduce the likelihood of including individuals whose prescriptions may have been influenced by non-medical or recreational motives. Although all patients were medically qualified for cannabis use under national guidelines, the restriction to patients ≥ 30 years was intended to enhance the clinical validity of the sample, as recreational use is statistically more prevalent in younger populations.
2. Referred to the cannabis clinic by their physician from various medical disciplines for consideration of a medical cannabis license.
3. Qualified for medical cannabis use without contraindications, according to the Israeli Ministry of Health guidelines (YAKAR). These guidelines specify that patients must not have:
  Active psychosis or a history of psychotic episodes.
  Anxiety disorders.
  Significant family history of psychiatric disorders, especially in patients under 30 years of age.
  A history of substance abuse.
  Current pregnancy.

### Exclusion criteria

Patients referred to the cannabis user clinic were excluded from the study if they were found ineligible for medical cannabis use due to the presence of any contraindications as outlined by the Ministry of Health or if they chose not to proceed with cannabis treatment.

### Measurements and data collection

Data were retrospectively collected from medical records and the clinic’s patient registry. The following information was recorded:

- Demographic data, including age, sex, familial status, and number of children.
- Indications for medical cannabis prescription - disease classifications were taken directly from patient records, categorised by primary medical condition (neurological, rheumatological, gastroenterological). Patients with comorbidities were classified based on the primary indication for which medical cannabis was prescribed.
- Details of cannabis prescription, including monthly dosage (in grams), THC/CBD ratio, cannabis cultivated variety, and consumption method (oil vs. smoking).

Data were collected from the electronic patient records, including Chameleon™ (Elad Group, Israel) and the Medical Cannabis Unit registry (YAKAR™ system).

The primary objective of this study was to describe and compare cannabis prescription patterns between different indications for medical cannabis.

A secondary objective was to assess potential differences in these same aspects of cannabis prescription patterns across various demographic factors, including age, sex, number of children, and familial status.

### Statistical analysis

Descriptive statistics were used to summarise the data. The distribution of continuous variables was assessed visually using histograms and QQ plots, and further tested for normality using the Kolmogorov–Smirnov test. Normally distributed continuous variables are reported as means ± standard deviation (SD), while non-normally distributed variables are presented as medians with interquartile range (IQR). Categorical variables are summarised as frequencies and percentages (%). For each comparison, the specific statistical test used is reported, along with the corresponding *p*-value, in the results tables and text. Specifically, one-way ANOVA was used to compare normally distributed continuous variables across the three disease groups (neurological, rheumatological, and gastroenterological), while the Kruskal–Wallis test was employed for variables with skewed distributions. Comparisons between two independent groups were performed using either independent-samples t-tests (for normally distributed variables) or Wilcoxon rank-sum tests (for non-normally distributed variables). For categorical variables, chi-square tests were used depending on expected cell sizes. All tests were two-sided, and *p*-values < 0.05 were considered statistically significant. Analyses were conducted using SAS Software, Version 9.4.

## Results

The study included 263 patients, categorised into three groups based on their primary medical condition: 106 in the rheumatological group [68 (64%) with fibromyalgia, 25 (23%) with Rheumatoid arthritis, and 13 (13%) with other rheumatological diseases], 94 in the gastrointestinal group [69 (73%) with Crohn’s disease and 25 (27%) with ulcerative colitis], and 63 in the neurological group [27 (42%) with multiple sclerosis, 13 (20%) with chronic neuropathic pain, 12 (19%) with Parkinson’s disease, 9 (15%) with Alzheimer’s disease and 2 (4%) with epilepsy]. The demographic characteristics of the patients are summarised in Table 1.

**Table 1:** Demographic, clinical and laboratory characteristics

Variable	Rheumatological group (*n* = 106)	Gastrointestinal group (*n* = 94)	Neurological group (*N* = 63)	*P* value
Male^#^, *n* (%)	26 (24.53%)	48 (51.06%)	36 (57.14%)	*P* < 0.001
Age^^^, years (mean ± SD)	53.56 ± 13.88	41.24 ± 16.03	61.01 ± 16.63	*P* < 0.0001
Marital status				*P* = 0.097
Single, *n* (%)	7 (6.60%)	15 (15.96%)	3 (4.76%)	
Married, *n* (%)	42 (39.62%)	47 (50%)	19 (30.16%)	
Divorced, *n* (%)	9 (8.5%)	8 (5.32%)	3 (4.76%)	
Widowed, *n* (%)	3 (2.83%)	0 (0%)	3 (4.76%)	
THC percentage (mean ± SD)	11.65 ± 6.92	13.32 ± 6.70	12.31 ± 5.69	*P* = 0.2238
Monthly cannabis dosage, grams (mean ± SD)	18.6 ± 11.57	22.26 ± 13.60	21.33 ± 13.75	*P* = 0.0439
Consumption method				*P* = 0.018
Smoke, *n* (%)	63 (59.4%)	72 (76.6%)	24 (38.1%)	
Oil, *n* (%)	29 (27.3%)	20 (21.3%)	20 (31.75%)	

Categorical variables are presented as counts and percentages. Normally distributed continuous variables are presented as the mean ± standard deviation (SD). THC, delta-9-tetrahydrocannabinol; ^#^For categorical variables, chi-square tests were used; ^^^ Comparisons between two independent groups were conducted using independent-samples t-tests.

The proportion of males differed significantly across groups, with 57.14% of the neurological group being male compared to 51.06% in the gastrointestinal group and only 24.53% in the rheumatological group (*p* < 0.001). Patients in the neurological group were the oldest, with a mean age of 61.01 ± 16.63 years, while the gastrointestinal group had the youngest patients (41.24 ± 16.03 years) (*p* < 0.0001).

The study explored various aspects of cannabis prescription patterns, including dosage, THC percentage, type of strain, and method of consumption.

Significant differences were found in the monthly cannabis dosage. Patients in the gastrointestinal group received the highest mean monthly dose (22.26 ± 13.60 g), followed by the neurological group (21.33 ± 13.75 g) and the rheumatological group (18.6 ± 11.57 g) (*p* = 0.0439).

The THC content did not differ between groups, with the gastrointestinal group having the highest mean THC percentage (13.32 ± 6.70%), followed by the neurological group (12.31 ± 5.69%) and the rheumatological group (11.65 ± 6.92%). However, this difference was not statistically significant (*p* = 0.2238).

Smoking was the most common method of cannabis use across all groups, with the highest prevalence in the gastrointestinal group (76.6%), followed by the rheumatological (59.4%) and neurological (38.1%) groups. Conversely, oil consumption was more frequent in the neurological group (31.75%) than in other groups. Differences in consumption methods were statistically significant across the groups (*p* = 0.018).

Table 2 shows the correlation between demographic factors (sex, marital status) and cannabis prescription patterns:

**Table 2:** Correlation between demographic characteristics and Cannabis prescribed

Variable	Sex		*P*-value	Marital status				*P*-value
	Male (*N* = 94)	Female (*N* = 138)		Single (*N* = 24)	Married (*N* = 95)	divorced (*N* = 15)	Widowed (*N* = 5)	
THC percentage^ (mean ± SD)	14 ± 6.56	11.39 ± 6.48	0.003	14.13 ± 6.04	12.95 ± 6.65	14.87 ± 5.63	6.40 ± 5.90	0.6879
Cannabinoid percentage (mean ± SD)	6.78 ± 5.17	8.69 ± 5.91	0.0282	7.00 ± 5.08	7.59 ± 6.05	7.00 ± 5.63	12.60 ± 6.43	0.899
Amount of cannabis prescribed in grams (mean ± SD)	25.48 ± 15.15	17.32 ± 9.93	< 0.0001	21.25 ± 15.9	20.63 ± 10.8	28.0 ± 20.77	12.0 ± 4.47	0.7931
Consumption method^#^			*P* = 0.189					*P* = 0.156
Smoke, *n* (%)	68 (72.3%)	91 (65.9%)		19 (79.16%)	65 (68.42%)	13 (86.66%)	2 (40%)	
Oil, *n* (%)	23 (24.4%)	46 (33.33%)		5 (20.83%)	30 (31.58%)	2 (13.34%)	3 (60%)	

Categorical variables are presented as counts and percentages. Normally distributed continuous variables are presented as the mean ± standard deviation (SD). THC, delta-9-tetrahydrocannabinol; ^#^For categorical variables, chi-square tests were used; ^^^Comparisons between two independent groups were conducted using independent-samples t-tests

Male patients were prescribed higher amounts of cannabis (25.48 ± 15.15 g) compared to females (17.32 ± 9.93 g) (*p* < 0.0001). THC percentages were also higher in male patients (14 ± 6.56%) than in females (11.39 ± 6.48%) (*p* = 0.003).

Cannabis prescriptions were relatively consistent across marital status categories (*p* = 0.7931). Smoking was more frequently prescribed to male patients (72.3%), whereas a higher proportion of female patients were prescribed oil-based formulations (33.33%). However, this difference in administration route was not statistically significant (*p* = 0.189).

## Discussion

This study aimed to characterise and compare the patterns of medicinal cannabis use across different disease groups (neurological, rheumatological, and gastrointestinal) and examine the influence of demographic factors such as sex, age, and marital status on cannabis. Our findings provide valuable insights into the demographics of patients using medicinal cannabis, the characteristics of their cannabis prescriptions, and the different consumption methods employed across disease categories.

Gastrointestinal patients were prescribed the highest mean dose of cannabis, which may be at least partially explained by the anti-inflammatory properties of cannabis (especially in IBD). This observation has been documented in previous studies that demonstrate significant symptomatic relief among IBD patients (Kalaba and Ware 2022; Ahmed and Katz 2016). Although the difference in THC percentage across groups was not statistically significant, the observed variations in prescribed cannabis dosages and potential differences in cannabinoid ratios, including CBD content, suggest that both dosage and cannabinoid composition may influence symptom management and warrant further investigation.

The observed variability in consumption methods also underscores how clinical needs shape cannabis prescription patterns. Smoking was significantly more prevalent among gastrointestinal patients, while oil-based cannabis was favoured by those with neurological conditions, especially older patients (Cuttler et al. 2016). 

The younger age profile of patients with gastrointestinal conditions, compared to those with neurological disorders, may be associated with reduced stigma regarding cannabis smoking, potentially influencing their mode of consumption.

Pronounced sex-based prescribing differences observed in our cohort are broadly consistent with, yet al.so extend, existing literature on differential cannabinoid responses. Large survey data have repeatedly shown that men tend to consume higher-THC products and report greater analgesic or appetite-stimulating motives, whereas women favour CBD-rich formulations (Cuttler et al. 2016; Bruce et al. 2021). Recent pharmacokinetic studies provide a biological substrate for these behavioural patterns: women exhibit higher systemic exposure to CBD metabolites after oral dosing (MacNair et al. 2024). and higher plasma 11-COOH-THC levels after smoke exposure in pre-clinical models, implying slower cannabinoid elimination (Gazarov et al. 2023). Conversely, a metered-dose-inhaler trial that standardised dose and route reported no acute sex differences in efficacy, safety, or THC pharmacokinetics, suggesting that formulation and delivery method can attenuate inherent variability (Aviram et al. 2023). 

Taken together, our finding that male patients were prescribed higher total THC doses, whereas females received proportionally more CBD-dominant products, aligns with epidemiological and mechanistic data while adding real-world prescription evidence from a clinically diverse sample. These converging data underscore the need for sex-responsive prescribing algorithms that integrate pharmacokinetic profiling, symptom targets, and potential withdrawal risks to optimise efficacy and minimise adverse effects across genders.

Interestingly, familial status did not significantly influence cannabis usage. While this suggests that these social factors may not directly impact cannabis prescriptions, future research with larger cohorts is needed to explore potential underlying psychological or social drivers that could affect patient preferences.

### Clinical implications and future research

Our findings underscore the need for personalised cannabis treatments that take into account both the medical condition and individual demographic characteristics, such as sex. For instance, the higher cannabis doses and THC content observed among male patients suggest that men may have different treatment needs or thresholds for cannabis therapy compared to women. These differences highlight the importance of individualised cannabis regimens to optimise therapeutic outcomes and minimise side effects. Recent advances in pharmacogenomics underscore the need for precision medicine in medicinal cannabis, particularly through pre-treatment screening of key genetic markers. Variants in the CNR1 gene (encoding CB_1_ receptors) have been linked to differences in cannabis dependence and response (Babayeva and Loewy 2023). A large observational study of 600 chronic pain patients showed that polymorphisms in CNR1, TRPV1, ABCB1, and UGT2B7 significantly predicted analgesic response to cannabis; patients with favourable allele combinations experienced markedly greater pain (Poli et al. 2022). Additionally, CYP450 enzyme variants (e.g., in CYP2C9, CYP2C19, CYP3A4/5) have been shown to alter THC/CBD metabolism, influencing drug exposure, efficacy, and adverse effects (Wright et al. 2025). Collectively, these data support integrating pharmacogenetic and receptor profiling before initiating cannabinoid therapy to optimise dosing, minimise side effects, and identify likely responders, thus advancing evidence-based, personalised cannabinoid prescribing.

Additionally, the preference for smoking over oil-based cannabis among gastrointestinal patients raises questions about the potential long-term effects of smoking on respiratory health. Clinicians should consider alternative methods of cannabis administration, especially for patients with chronic conditions or comorbidities that may be exacerbated by smoking.

This study also highlights the complexity of managing cannabis use across different medical conditions, with variations in dosage, THC content, and method of consumption. The lack of significant differences in THC percentage between disease groups suggests that the choice of cannabis formulation may be more influenced by individual patient factors rather than the condition itself. Future research should focus on identifying more precise clinical guidelines for cannabis prescription based on disease-specific needs, as well as exploring the long-term effects of cannabis use on health outcomes across different patient populations.

### Limitations

Several limitations should be considered when interpreting the results of this study. First, the study’s retrospective nature may introduce bias in data collection, and the lack of randomisation limits the ability to infer causality. Second, fibromyalgia is not classified as an inflammatory disease and is increasingly recognised as a central nervous system disorder; it is often managed by rheumatologists in clinical settings. This may have influenced the clinical categorisation in our study. Third, this study is based on prescription records, which reflect physician-authorised recommendations for medical cannabis use rather than confirmed patient consumption. While prescriptions provide important insights into clinical decision-making, they do not capture actual usage patterns, dosage adherence, or route of administration. As such, we acknowledge that relying solely on prescription data limits our ability to assess real-world consumption behaviours. Additionally, one important limitation of this study is the small sample size within several clinical subgroups, which may affect the reliability and generalizability of specific findings. In particular, some cell sizes were small, limiting the statistical power to detect differences and increasing the potential for type II errors. Future studies with larger, more diverse cohorts are needed to validate these findings and provide more robust conclusions.

## Conclusion

In conclusion, this study provides important insights into the characteristics of medicinal cannabis use among patients with different medical conditions and demographic backgrounds. While patterns of cannabis use vary between disease groups, particularly in terms of dosage, THC content, and consumption method, demographic factors such as sex also play a significant role in shaping cannabis therapy. Personalised approaches to cannabis prescribing, informed by both clinical and demographic considerations, are essential to optimising treatment outcomes. Further research is needed to establish more precise guidelines for medicinal cannabis use, taking into account the complex interplay between medical indications and individual patient factors.

## Data Availability

Data will be available by request.
